# Apps for People With Rheumatoid Arthritis to Monitor Their Disease Activity: A Review of Apps for Best Practice and Quality

**DOI:** 10.2196/mhealth.6956

**Published:** 2017-02-21

**Authors:** Rebecca Grainger, Hermaleigh Townsley, Bonnie White, Tobias Langlotz, William J Taylor

**Affiliations:** ^1^ Rehabilitation Teaching and Research Unit Department of Medicine University of Otago Wellington Wellington New Zealand; ^2^ Hutt Hospital Hutt Valley District Health Board Lower Hutt New Zealand; ^3^ Department of Medicine University of Otago Wellington Wellington New Zealand; ^4^ Department of Information Science University of Otago Dunedin New Zealand

**Keywords:** mHealth, mobile applications, telemedicine, self-management, rheumatoid arthritis

## Abstract

**Background:**

Rheumatoid arthritis (RA) is a chronic inflammatory arthritis requiring long-term treatment with regular monitoring by a rheumatologist to achieve good health outcomes. Since people with RA may wish to monitor their own disease activity with a smartphone app, it is important to understand the functions and quality of apps for this purpose.

**Objective:**

The aim of our study was to assess the features and quality of apps to assist people to monitor their RA disease activity by (1) summarizing the available apps, particularly the instruments used for measurement of RA disease activity; (2) comparing the app features with American College of Rheumatology and European League against Rheumatism (ACR and EULAR) guidelines for monitoring of RA disease activity; and (3) rating app quality with the Mobile App Rating Scale (MARS).

**Methods:**

Systematic searches of the New Zealand iTunes and Google Play app stores were used to identify all apps for monitoring of RA disease activity that could be used by people with RA. The apps were described by both key metadata and app functionality. App adherence with recommendations for monitoring of RA disease activity in clinical practice was evaluated by identifying whether apps included calculation of a validated composite disease activity measure and recorded results for future retrieval. App quality was assessed by 2 independent reviewers using the MARS.

**Results:**

The search identified 721 apps in the Google Play store and 216 in the iTunes store, of which 19 unique apps met criteria for inclusion (8 from both app stores, 8 iTunes, and 3 Google Play). In total, 14 apps included at least one validated instrument measuring RA disease activity; 7 of 11 apps that allowed users to enter a joint count used the standard 28 swollen and tender joint count; 8 apps included at least one ACR and EULAR-recommended RA composite disease activity (CDA) measure; and 10 apps included data storage and retrieval. Only 1 app, Arthritis Power, included both an RA CDA measure and tracked data, but this app did not include the standard 28 tender and swollen joint count. The median overall MARS score for apps was 3.41/5. Of the 6 apps that scored ≥4/5 on the overall MARS rating, only 1 included a CDA score endorsed by ACR and EULAR; however, this app did not have a data tracking function.

**Conclusions:**

This review found a lack of high-quality apps for longitudinal assessment of RA disease activity. Current apps fall into two categories: simple calculators primarily for rheumatologists and data tracking tools for people with RA. The latter do not uniformly collect data using validated instruments or composite disease activity measures. There is a need for appropriate, high-quality apps for use by rheumatologists and patients together in co-management of RA.

## Introduction

Rheumatoid arthritis (RA) is a systemic inflammatory disease characterized by a symmetrical polyarthritis due to immune-mediated inflammation of synovial tissue [1,2]. The symptoms include painful and swollen joints with fatigue and morning stiffness. Uncontrolled polyarthritis can damage cartilage and bone [1,2]. Therefore, long-term treatment with disease-modifying antirheumatic drugs to control inflammation is required, ideally under the supervision of a rheumatologist [1]. The disease course can be unpredictable, with periods of relatively lower disease activity interspersed with flare-ups. Treatment response is also unpredictable with marked individual variation in drug effectiveness or adverse effects, and there are changes in efficacy over time. Regular follow-up and monitoring of patient disease activity to guide treatment is required to achieve RA remission or low disease activity state [3] and patient-centered care is important in the optimal management of RA [4]. Guidelines recommend that rheumatologist assessment of RA disease activity should include some or all of the validated measures of disease activity or patient physical function, and a composite disease activity measure, such as the Disease Activity Score including 28 joints (DAS28) [3,5].

Mobile health (mHealth) is a rapidly growing area of health care delivery, where mobile devices, particularly via mobile apps on smartphones, are used to support medical and public health practice [6]. mHealth apps may be useful tools for patient self-management, as well as for facilitating improved communication between patients and health care providers [7]. In the United States, over two-thirds of adults own a smartphone [8]. mHealth is therefore increasingly accessible, and there are now numerous health-related smartphone apps available [9,10]. For chronic conditions such as RA, mHealth may provide a way for patients to become more actively involved in their disease management. In a Portuguese study, 86 of 100 people with RA agreed that a smartphone app for RA self-management would be useful [11]. Younger age, current smartphone ownership, and use of email, Internet, and short messaging services were all associated with willingness to use apps for RA self-management. A small Japanese study reported that patient self-reported disease activity data using validated instruments correlated well with rheumatologist-assessed RA disease activity [12]. Furthermore, there is some evidence that mHealth interventions such as smartphone apps may improve outcomes for people with other chronic diseases [7,13,14].

With an increasing number of mHealth apps available, potential users need to be able to determine the quality of health-related apps. A systematic literature review demonstrated that many health apps did not adhere to evidence-based guidelines and did not involve medical experts during development [15]. When assessing app quality, users currently have little information beyond the description of the app and a star rating. Therefore, they may rely on an app that is not based on best practice or medical evidence and could even be unsafe. As mHealth apps become pervasive, it is important that users can make informed decisions about the apps they use.

Recently, the Mobile App Rating Scale (MARS) was developed as a tool for classifying and rating the quality of mHealth apps [16]. The 23 items in the MARS were identified from a review of existing criteria for rating app quality. Each item was rated on a 5-point scale (1=inadequate, 2=poor, 3=acceptable, 4=good, and 5=excellent) with descriptors provided for each anchor rating. The MARS grouped the items in 4 categories: engagement (5 items), functionality (4 items), aesthetics (3 items), and information quality (7 items), as well as 1 subjective quality scale (4 items). The MARS was scored with a mean for each of the categories and an overall mean score. The MARS demonstrated good internal consistency and inter-rater reliability and provided a reliable method to rate and compare mobile apps [16,17].

Since mHealth apps have the potential to allow people with RA to monitor their RA disease activity, it is important to assess the features and quality of smartphone apps currently available. Apps that collect disease activity data using validated disease activity instruments may be useful in facilitating management with a rheumatologist by measuring medically credible RA activity between visits and potentially enabling some care to be provided via telehealth [18,19].

The objective of this study was to determine whether there are existing high-quality apps for monitoring RA disease activity that use validated, recommended measurement instruments, have functionality to share these data with the treating rheumatologist, and are currently available for public use. The specific aims of this review were to assess the features and quality of apps designed to assist people to monitor their RA disease activity by: (1) summarizing the available apps and the key features, particularly the instruments used for measurement of RA disease activity; (2) comparing the app features with guidelines for monitoring of RA disease activity; and (3) rating app quality according to the MARS. This will enable informed decisions about app use and may identify gaps or deficiencies in the mHealth apps for RA disease activity monitoring currently available.

## Methods

### App Identification

A systematic search of the New Zealand iTunes and Google Play stores was conducted on April 1, 2016, to identify all potentially relevant apps. The search was conducted following the Preferred Reporting Items for Systematic Reviews and Meta-Analyses (PRISMA) guidelines for systematic reviews [20]. Search terms included “arthritis” OR “rheumatoid” OR “RA” OR “rheumatoid arthritis” OR “rheumatic.” The app store description of each identified app was read and compared with the inclusion and exclusion criteria. Apps were included if they were: (1) a smartphone-based app; (2) capable of running on Android or iOS operating systems; (3) in English language; (4) useful for people with RA or to assist clinical care of people with RA; and (5) available for download in the New Zealand app store (iTunes or Google Play). Apps were excluded if: (1) a condition other than RA was targeted; (2) app content was for information, education, or reference only (ie, no data entry); (3) the app included only treatment algorithms; or (4) it was explicitly only for clinician use. When an app was found in both the Google Play and iTunes store, both versions were included so any differences between operating systems could be identified. Android apps (New Zealand Google Play store) were downloaded and tested using 2 Samsung Galaxy J1 Ace phones equipped with Android version 5.1.1. iOS apps (New Zealand iTunes store) were downloaded and tested using iPhones (4s and 6) with iOS 9.1 installed.

Since the New Zealand app stores may not include all potentially relevant apps, the United States, the United Kingdom, Australia, and Canada iTunes stores were also searched for eligible apps by conducting the search using the terms “rheumatoid arthritis” on the website fnd.io [21]. “Rheumatoid arthritis” was used as the sole search term, as this returned almost all apps found in the main search and did not identify any additional apps.

### Data Extraction

The following data about all apps were recorded: app name, platform (Android, iOS), developer, current version, size, cost, number of installs, and user star ratings. Functional features were noted descriptively.

### Comparison of Apps to Rheumatoid Arthritis Management Recommendations

App adherence with relevant recommendations for monitoring of RA disease activity in clinical practice from the American College of Rheumatology (ACR) and the European League Against Rheumatism (EULAR) was evaluated [3,5]. This was determined by operationalizing the recommendations and determining whether present or not present in each app (Table 1).

**Table 1:** Recommendations from the American College of Rheumatology (ACR) and the European League Against Rheumatism (EULAR) for rheumatoid arthritis (RA) disease activity monitoring.

Organization	Recommendation	Instruments required	Composite disease activity score	Adherence by app present if:
ACR^a^ [5]	The use of ACR-recommended validated composite measures of disease activity is needed to treat to target in clinical practice.	PtG^c^, PhG^d^, HAQ^e^, 28TJC^f^, 28SJC^g^, CRP^h^, ESR^i^	Patient-driven tools: PAS^j^; PAS-II^k^, RAPID-3^l^ Patient + provider: CDAI^m^ Patient + provider + laboratory: DAS28^n^, SDAI^o^	One or more of composite disease activity scores were calculated by the app, using the validated component instruments.
EULAR^b^ [3]	The use of validated composite measures of disease activity, which include joint assessments, is needed in routine clinical practice to guide treatment decisions.	PtG, PhG, HAQ, 28TJC, 28SJC, CRP, ESR	PAS, PAS-II, RAPID-3, SDAI, CDAI, and DAS28 (CRP or ESR)	One or more of composite disease activity scores were calculated by the app, using the validated component instruments.
	Measures of disease activity must be obtained and documented regularly, as frequently as monthly for patients with high or moderate disease activity or less frequently (such as every 6 months) for patients in sustained low-disease activity or remission.			Users were able to record disease activity on multiple occasions with data recorded and retrievable within the app.

^a^ACR: American College of Rheumatology.

^b^EULAR: European League Against Rheumatism.

^c^PtG: patient global assessment of disease activity.

^d^PhG: physician global assessment of disease activity.

^e^HAQ: health assessment questionnaire.

^f^28TJC: 28 tender joint count.

^g^28SJC: 28 swollen joint count.

^h^CRP: C-reactive protein.

^i^ESR: erythrocyte sedimentation rate.

^j^PAS: patient activity scale.

^k^PAS-II: patient activity scale II.

^l^RAPID-3: routine assessment of patient index data.

^m^CDAI: clinical disease activity index.

^n^DAS28: disease activity index.

^o^SDAI: simple disease activity index.

### App Rating Using the MARS

All apps were rated by two independent reviewers (HT and BW) using the MARS [16]. Before app assessment, the two reviewers discussed the use of the MARS in the context of apps for people with RA. The target group was determined to be “all people with RA aged 18 years or older; some familiarity with smartphone technology.” As recommended by the developers of the MARS, the reviewers considered all items of the MARS and confirmed that all were applicable to apps for RA, and that no additional app-specific items were required [16]. The reviewers also viewed the training video developed by Stoyanov et al.

Before assessing all the apps identified in the search, both reviewers assessed and discussed an excluded app to ensure shared understanding of the MARS items and process. The reviewers then independently rated all apps using the MARS. Before scoring each app, the reviewers used each app for at least ten minutes to gain an adequate understanding of the app functionality. Apps were tested on April 11, 2016, using the app version downloaded on April 1, 2016. Any issues or uncertainties about specific apps were discussed, and consensus was reached.

Scores were calculated for each MARS item, along with a total mean score. The mean score from two reviewers was calculated. No apps had been tested in clinical studies. Therefore, MARS item 19 “evidence base” was excluded from calculations. Inter-rater reliability of the MARS subscales and total quality score were calculated using the intraclass correlation coefficient (ICC) in SPSS Version 20.0 (IBM Corp; 2-way random-effects model of absolute agreement between single ratings).

## Results

### Systematic Search for Apps

**Figure 1:**
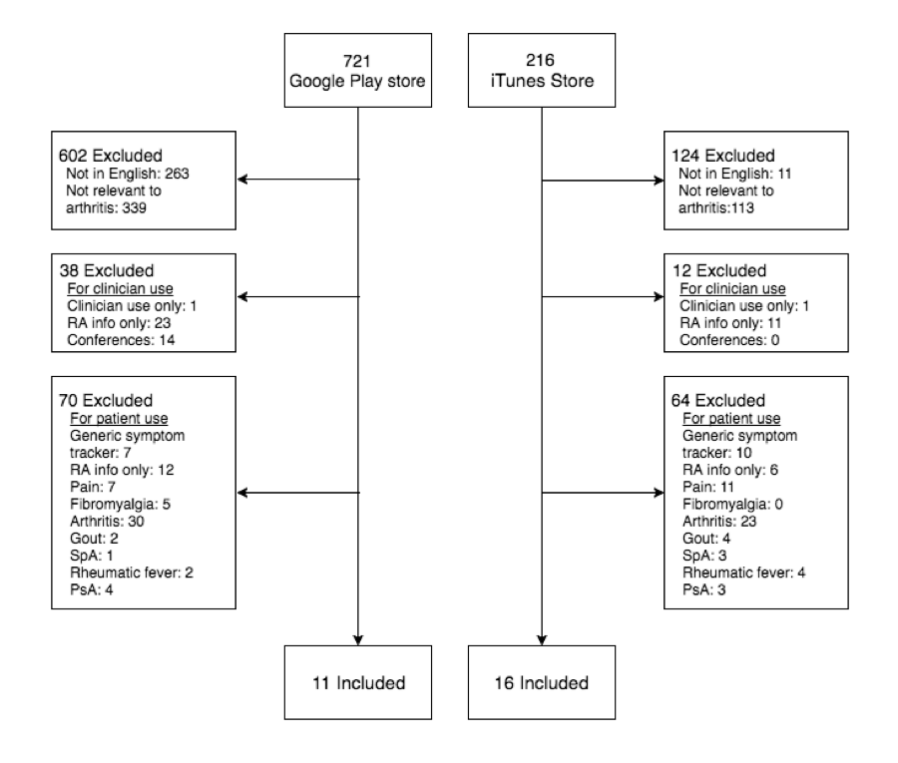
Flow diagram of systematic search and selection of app from Google Play and iTunes stores.

The search retrieved 721 Android apps from the Google Play store. Of these, 710 were excluded, leaving 11 apps for analysis (Figure 1). A total of 216 iOS apps were retrieved from the iTunes app store. After exclusion of 200 apps, 16 apps remained for analysis. No further apps were found in the fnd.io search of the United States, the United Kingdom, Australia, and Canada iTunes stores. As 8 apps were available in both operating systems, a total of 19 different apps were included, of which 18 were free apps (Rheumatoid Arthritis Diary was available for NZD $6.39 for Android and NZD $6.49 for iOS).

### Characteristics and Functions of Included Apps

The information on app platform, developer, version, and size are shown in Table 2. Since no apps had different functionality between operating systems, the apps are presented only once in Tables 2-6. The app description, target user (as derived from the app store description), Android installs, and Android star rating are shown in Table 3. None of the iOS apps included had the minimum of 5 reviews from users in New Zealand required on the New Zealand iTunes store before a star rating is provided. Table 4 shows joint count data entry and main functionality in the apps. Eleven apps allowed users to enter a joint count, either by selecting joints on a homunculus (n=4) or by entering the number of joints (n=7). Seven of these apps included the standard 28 swollen and tender joint count and primarily functioned as disease activity measure calculators, with no capacity to store or track data. The remaining 4 apps with other joint counts (Cliexa-RA, myRA, RheumaTrack RA, and RAPA) all had additional patient-focused functions, such as recording fatigue, and storage and tracking of imputed data. Fourteen apps included calculation of a RA disease activity measure. Six apps allowed export of patient data, including via email (n=5), spreadsheet (n=2), or to a website (n=2).

**Table 2:** Operating system, developer, version, and size of included apps.

App	Operating system	Developer	iOS version^a^	iOS size (MB)	Android version^a^	Android size (MB)
Arthritis Power	iOS	Jeffrey Curtis	1.2.1	3.5	–	–
Cliexa-RA^b^	iOS	CN4CE, LLC	1.01	12.6	–	–
DAS Calculator for Rheumatologists	iOS	Greg Fiumara	1.13	0.596	–	–
DAS28^c^-Rheumatoid Arthritis	Android	Tantor Systems	–	–	2.5	0.52
DAS28 Calculator	iOS	Rheumatology LMU	2.1	0.654	–	–
DAS28 Calculator	Android	Owl Studios	–	–	2.1	1.4
DAS28/ACR-EULAR criteria	iOS	Keiji Matsui	3.1	0.2	–	–
DAS28 Free	Android	Esdras Beleza de Noronha	–	–	1.0.	0.762
myRA	iOS	Crescendo Bioscience Inc	1.7	3.3	–	–
myRAteam	Android, iOS	MyHealthTeams Inc	10.10.0	4.7	10.10.a	1.8
RA Helper	Android, iOS	Modra Jagoda	2.1	4.9	1.2	3.4
RAISE	Android, iOS	Publicis Development-Arthritis Ireland	1.0.3	16.7	1.0.3	7.3
RAPA^d^	Android, iOS	Jacsomedia Ltd	1.0.	3.7	4	2.0
Rheuma Helper	Android, iOS	Modra Jagoda	2.3	3.3	2.3	2.7
Rheumatoid Arthritis Diary	Android, iOS	cellHigh	1.6.7	15	1.6.4	2.6
Rheumatoid Arthritis Patient Companion	iOS	Point of Care	3.27.6	10.4	–	–
RheumaTrack RA	Android, iOS	Axovis GmbH	2.0.7	10.5	2.0.9	5.6
RheumInfo HAQ^e^-II Calculator	iOS	Bitcurve Systems	1	1.2	–	–
TRACK and REACT	Android, iOS	Arthritis Foundation	1.8	5.6	1.3	2.2

^a^version available on April 1, 2016.

^b^RA: rheumatoid arthritis.

^c^DAS28: disease activity score 28 joints.

^d^RAPA: RA Patient Application.

^e^HAQ: health assessment questionnaire.

**Table 3:** Description, target user, Android installs, and star rating of included apps.

App	Description	Target user	Android installs^a^ (×10^4^)	Android star rating
Arthritis Power	Input data to monitor disease	People with arthritis	–	–
Cliexa-RA^b^	Input data to monitor disease	People with RA	–	–
DAS Calculator for Rheumatologists	DAS28 calculator	Clinicians	–	–
DAS28^c^-Rheumatoid Arthritis	DAS28 calculator	Clinical practice or trials	10-50	4.1
DAS28 Calculator	DAS28 calculator	Not stated	–	–
DAS28 Calculator	DAS28 calculator	Not stated	1-5	3.4
DAS28/ACR-EULAR criteria	Various calculators	Not stated	–	–
DAS28 Free	DAS28 calculator	Clinicians	1-5	3.6
myRA	Input data to monitor disease	People with RA	–	–
myRAteam	Social media for people with RA	People with RA	1-5	4.5
RA Helper	Input data to monitor disease	People with RA	1-5	4.3
RAISE	Patient monitor exercise and pain levels	People with RA	0.1-0.5	2
RAPA^d^	Input data to monitor disease	People with RA	0.1-0.5	4.7
Rheuma Helper	Calculator with info for rheumatologists	Clinicians	0.1-0.5	4.4
Rheumatoid Arthritis Diary	Input data to monitor disease	People with RA	0.05-0.1	–
Rheumatoid Arthritis Patient Companion	Input data to monitor disease	People with RA	–	–
RheumaTrack RA	Input data to monitor disease	People with RA	10-50	4.2
RheumInfo HAQ^e^-II Calculator	HAQII calculator	Not stated	–	–
TRACK and REACT	Patient monitor exercise and pain levels	People with RA	10-50	3.3

^a^Install data available only for Android in Google Play store, as of search date on April 1, 2016.

^b^RA: rheumatoid arthritis.

^c^DAS28: disease activity score 28 joints.

^d^RAPA: RA Patient Application.

^e^HAQ: health assessment questionnaire.

**Table 4:** Joint count and other functionality of included apps.

App	Joint count		Data entered	Composite disease activity measure	Other functions
	Number	Measure			
Arthritis Power			Medication, sleep, exercise, fatigue	✔	Email data, export to website
Cliexa-RA^a^	26	S^e^, T^f^	Medication	✔	
DAS Calculator for Rheumatologists	28	S, T		✔	
DAS28^b^-Rheumatoid Arthritis	28	S, T		✔	
DAS28 Calculator	28	S, T		✔	
DAS28 Calculator	28	S, T		✔	
DAS28 Free	28	S, T		✔	
DAS28/ACR-EULAR criteria	28	S, T		✔	
myRA	44	P^g^	Medication, lab^e^		Reminders, email data, RA info^f^
myRAteam			Free text		Within app social media function
RA Helper			Medication, lab		Reminders
RAISE			Pain, activity,		Email data, RA info
RAPA^c^	28	S	Work, fever	✔	RA info
RheumaHelper	28	S, T		✔	RA info
Rheumatoid Arthritis Diary			Medication, lab, pain, symptoms, activity, triggers, sleep, mood		Email data, export to spreadsheet
Rheumatoid Arthritis Patient Companion			Medication, lab, mood, symptoms, activity	✔	Reminders, share data with clinician, RA info
RheumaTrack RA	52	S, P	Medications, morning stiffness, work, exercise, infection	✔	Email data, export to spreadsheet
RheumInfo HAQ^d^-II Calculator				✔	
TRACK and REACT			Medication, stiffness, joint function, social, exercise, energy	✔	Export to website

^a^RA: rheumatoid arthritis.

^b^DAS28: disease activity score 28 joints.

^c^RAPA: RA Patient Application.

^d^HAQ: health assessment questionnaire.

^e^S: swollen.

^f^T: tender.

^g^P: pain.

^e^Lab: laboratory data.

^f^RA info: rheumatoid arthritis information.

### Comparison of Apps to Rheumatoid Arthritis Management Recommendations

App inclusion of the component measurement instruments, composite disease activity measures calculated, and app functionality to record and retrieve data over time (as recommended by ACR and EULAR [3,5]) are shown in Table 5. Eight apps included at least one recommended composite measure of RA disease activity. Only 1 of these 8 apps provided the formulae for calculation of the composite disease activity measures (RheumaHelper), which were confirmed to be the correct formulae. Ten apps included a function allowing data to be recorded and retrieved. One app, Arthritis Power, included both 1 composite disease activity measure and allowed data recording and retrieval, but this app did not have functionality to record a 28 tender or swollen joint count.

**Table 5:** App inclusion of the rheumatoid arthritis (RA) activity measures and component measurement instruments.

App	ACR and EULAR-endorsed instruments or laboratory measures	ACR and EULAR-recommended composite disease activity measure	Equation provided for composite disease activity measure	Allows users to record and retrieve disease activity data on multiple occasions
Arthritis Power	PtG^c^, Pain VAS^d^	RAPID-3^j^	No	History, graph
Cliexa-RA^a^	PtG, ESR^f^			History, graph
DAS Calculator for Rheumatologists	PtG, CRP^g^, 28SJC^h^, 28TJC^i^	DAS28^m^	No	
DAS28-Rheumatoid Arthritis	PtG, ESR, CRP, 28SJC, 28TJC	DAS28	No	
DAS28 Calculator	PtG, CRP, 28SJC, 28TJC	DAS28	No	
DAS28 Calculator	PtG, 28SJC, 28TJC	DAS28	No	
DAS28 Free	PtG, CRP, 28SJC, 28TJC	DAS28	No	
DAS28/ACR-EULAR criteria	PtG, Pain VAS, ESR, CRP	DAS28, CDAI^l^, SDAI^k^	No	
myRA	ESR, CRP			History, graph
myRAteam				History
RA Helper	ESR, CRP			History, graph
RAISE				History, graph
RAPA^b^	Pain VAS			History, graph
RheumaHelper	PtG, CRP, 28SJC, 28TJC	DAS28, CDAI, SDAI	Yes	
Rheumatoid Arthritis Diary	ESR, CRP			History, graph
Rheumatoid Arthritis Patient Companion				History, graph
RheumaTrack RA				History, graph
RheumInfo HAQ-II Calculator	HAQ^e^			
TRACK and REACT	Pain VAS			History, graph

^a^RA: rheumatoid arthritis.

^b^RAPA: RA Patient Application.

^c^PtG: patient global assessment of disease activity.

^d^VAS: visual analog scale.

^e^HAQ: health assessment questionnaire.

^f^ESR: erythrocyte sedimentation rate.

^g^CRP: C-reactive protein.

^h^28SJC: 28 swollen joint count.

^i^28TJC: 28 tender joint count.

^j^RAPID-3: routine assessment of patient index data.

^k^SDAI: simple disease activity index.

^l^CDAI: clinical disease activity index.

^m^DAS28: disease activity index 28 joint count.

### MARS Rating of Apps

MARS ratings for included apps are shown in Table 6. The ICC for MARS ratings was greater than or equal to 0.69 for all MARS sections. For overall MARS ratings, the ICC was .93 (95% CI 0.76-0.98) for Android apps and .82 (95% CI 0.55-0.94) for iOS apps, confirming good inter-rater reliability. The overall MARS scores for the apps ranged from 1.98 to 4.62, indicating large variation in the quality of apps. Engagement (1.6-4.8) and aesthetics (1.17-4.67) showed greatest variability. Of the 6 apps that scored ≥4/5 on the overall MARS rating, only 1 (RheumaHelper) included a composite disease activity score endorsed by ACR and EULAR, but this app did not have a data tracking function. Of the other 5 apps scoring ≥4/5 on the overall MARS rating (myRA, RAISE, myRAteam, Rheumatoid Arthritis Patient Companion, and RheumaTrack RA), 2 allowed entry of CRP and ESR but no other validated RA disease activity instruments were included in these apps. Arthritis Power, the only app that included an ACR and EULAR–recommended composite disease activity score and tracked results had an overall MARS score of 3.41.

**Table 6:** Mean mobile app rating scale (MARS) ratings of included apps.

App name	MARS^a^ categories															
	Engagement (5 items)		Functionality (4 items)			Aesthetics (3 items)			Information (7 items)			Subjective (4 items)			Overall MARS mean score	
	Android	iOS	Android	iOS	Android		iOS	Android		iOS	Android		iOS	Android		iOS
Arthritis Power		3.60		3.13			3.33			3.58			2.25			3.41
Cliexa-RA^b^		3.20		4.00			3.83			3.42			2.38			3.61
DAS Calculator for Rheumatologists		3.00		5.00			4.00			3.40			2.63			3.85
DAS28^c^-Rheumatoid Arthritis	1.70		3.25		2.17			3.25			1.50			2.59		
Das28 Calculator		2.70		4.38			3.50			3.35			2.13			3.48
Das28 Calculator	1.60		3.00		1.17			2.00			1.25			1.94		
DAS28/ACR-EULAR criteria		1.70		3.75			1.33			2.33			1.25			2.28
DAS28 Free	2.20		4.38		3.00			2.92			2.25			3.13		
myRA		4.80		4.50			4.67			4.50			4.38			4.62
myRAteam	4.50	4.50	3.63	3.63	4.00		4.00	4.33		4.33	4.25		3.75	4.11		4.11
RA Helper	2.20	2.20	4.00	4.00	4.17		4.17	2.42		2.42	1.50		1.50	3.20		3.20
RAISE	4.00	4.00	4.38	4.38	4.34		4.34	4.25		4.25	3.25		3.25	4.24		4.24
RAPA^d^	2.40	2.40	3.00	3.00	2.67		3.00	3.34		3.34	1.75		1.75	2.85		2.93
Rheuma Helper	3.60	3.60	4.88	4.88	4.33		4.33	4.25		4.25	4.00		4.00	4.26		4.26
Rheumatoid Arthritis Diary	3.60	3.60	2.50	2.50	1.83		1.83	3.08		3.08	2.50		2.50	2.75		2.75
Rheumatoid Arthritis Patient Companion		4.40		3.63			4.00			4.58			3.13			4.15
RheumaTrack RA	3.80		4.63		4.50			4.42			4.50			4.34		
RheumInfo HAQ^e^-II Calculator		2.20		4.13			2.67			2.80			1.38			2.95
TRACK and REACT	3.90	3.90	2.75	2.75	2.83		2.83	3.75		3.75	2.63		2.63	3.31		3.31
Reliability of MARS rating																
Two-way random effects ICC using absolute agreement between single ratings (95% CI)	0.93 (0.77- 0.98)	0.92 (0.79-0.97)	0.87 (0.60- 0.96)	0.83 (0.57- 0.94)	0.91 (0.69-0.97)		0.87 (0.47.-0.96)	0.83 (0.51- 0.95)		0.83 (0.51- 0.94)	0.80 (0.43-0.94)		0.69 (0.15-0.89)	0.93 (0.76- 0.98)		0.82 (0.55-0.94)

^a^MARS: mobile app rating scale.

^b^A: rheumatoid arthritis.

^c^DAS28: disease activity score 28 joints.

^d^RAPA: RA Patient Application.

^e^HAQ: health assessment questionnaire.

## Discussion

### Principal Findings

This review of apps for monitoring disease activity in people with RA showed that there are broadly two categories of apps available: apps for calculation of validated disease activity measures and those for people with arthritis to track symptoms. Many symptom-tracking apps did not use validated instruments. Apps that focused on calculations of a disease activity measure tended to only perform that function. One app, myRAteam, provided an environment in which people with RA could connect and share updates about their symptoms. Other less commonly encountered app functions included setting reminders and information sharing with a clinician, either via email or through a linked app. The latter is essential for an app to facilitate telehealth. Six apps allowed email or sharing of data, and only 1 of these apps provided a mechanism for sharing specifically with a clinician. This indicates a lack of apps suitable for large-scale telehealth management of RA.

Only one app, Arthritis Power, included both a symptom-tracking function and calculation of an ACR and EULAR-recommended composite measure of RA disease activity. However, Arthritis Power did not include a joint count function. Some apps appear to perform both functions, but include an incorrect version of a disease activity measure, for example, a 28 swollen joint count without a tender joint count (eg, RAPA). Overall, 14 apps provided a composite disease activity score, but only 8 apps used the correct component instruments to calculate the composite disease activity measure and therefore provided an ACR- and EULAR–recommended composite measure. A common reason for measures to not meet the latter criterion was the use of a joint count that did not specify tender and swollen as the joint abnormalities of interest or did not count the 28 joints required for a DAS-28. Some apps recording joint symptoms may be useful for people with RA to monitor their symptoms, but could not be used in a remote monitoring telehealth care service. People with RA wishing to monitor their own symptoms should be encouraged to choose apps, which use validated instruments and have a tracking function, such as Arthritis Power.

There were no apps that scored ≥4/5 on the overall MARS and included all ACR and EULAR endorsed disease activity instruments. This could be because apps are designed with either people with RA or rheumatologists as target users where patients do not usually perform joint counts and doctors would not usually need to store patient data in a mobile phone. The MARS scores had a wide range indicating highly variable quality of apps in terms of user experience. Future app development should occur with cooperation between software developers and key stakeholders. Software developers should optimize user experience in collaboration with people with RA, while doctors can ensure app adherence with best-practice evidence-based medicine. Item 19 of the MARS, “evidence base,” was excluded from all calculations because no apps had been studied in clinical trials, as specified by Stoyanov et al [16]. Therefore any future apps developed for RA disease activity monitoring should be assessed in clinical trials to determine the impact on clinical outcomes for people with RA and cost-effectiveness and undergo external quality review [22].

### Limitations

This study had a number of limitations. Only apps available in New Zealand app stores and in English language were included. An app for patient-led monitoring of RA disease activity has been developed in Japanese, which includes ACR and EULAR recommended instruments and diseases activity measures [12]. However, a preliminary search of the iTunes stores of 4 other English-speaking countries with the term “rheumatoid arthritis” suggested that the search of the New Zealand app stores has captured all relevant apps in the English language.

App quality was assessed using the MARS. The MARS is a recently developed tool and has not been extensively validated. However, it has now been used in several other app evaluations [17,23,24], and as in this study, the MARS has consistently proven good inter-rater reliability. App quality was also assessed by considering whether apps complied with ACR and EULAR RA management recommendations. There may have been other criteria that could have been used to assess app quality. Assessment of data security is not included in the MARS but is one commonly considered criterion of health software quality not included in this study [25]. Data security considerations are of utmost importance but will need to be considered within the regulatory requirements of the country in which the app is being used. The integration of health behavior theory concepts into app design and function, which has been used as a measure of quality, was also not considered in this study [26,27].

The recommended RA composite disease activity (CDA) scores include those with exclusively patient-reported outcomes (eg, RAPID 3 and PAS) and those that combine patient-reported outcomes and physician-performed tender and swollen joint counts. Remote telehealth monitoring of disease activity for people with RA assumes either that disease activity is derived from patient-reported outcomes or that patient self-performed joint counts will provide sufficiently accurate assessments of RA disease activity. Patient-performed joint counts do correlate moderately with physician-performed joint counts [28]. However, further validation of the assumption that patient-performed joint counts will be sufficient for longitudinal measurement of RA disease activity is required.

### Comparison With Prior Work

The findings of this study suggest that currently available RA apps for RA disease activity monitoring are of variable quality and generally do not comply with RA management guidelines. Many other studies of health apps have found that most apps do not comply with evidence-based guidelines [29-31]. Like RA, inflammatory bowel disease requires ongoing management by a specialist physician and has a variable, unpredictable clinical course. A comprehensive analysis of apps for inflammatory bowel disease (IBD) identified that only 54% (14/26) provided a symptom-tracking function and only 19% (5/26) had medical input during app development [32]. Eight apps were specifically for providing information about IBD. When information about IBD included in these apps was compared with the minimum information set of 14 statements recommended to be shared with people with IBD, only 38% of these statements had complete coverage in the apps [32]. Similarly, a review of apps for people with gout, another common form of chronic arthritis, found only 1 of 6 relevant apps included all recommendations for patient-focused quality care of gout [33]. These studies suggest that the lack of a comprehensive, guideline-compliant app for RA is part of a wider paucity of high-quality health apps available.

The inclusion of persuasive principles, aimed to support positive behavioral change, has been considered as a measure of app quality in a recent systematic analysis of apps for chronic arthritis [34]. In the 28 assessed apps, a mean of only 5.8 of 37 persuasive principles per app was found with social support techniques (eg, social media, user forums) and sophisticated dialogue support techniques (eg, praise, rewards) largely absent. This suggests that the design process for future for RA should consider evidence-based persuasive techniques.

### Conclusions

This review indicated the lack of high-quality apps available to assist in the management of RA, particularly the longitudinal assessment of RA disease activity. Only 1 app of the 19 identified in this study had functionality to allow both calculation of a validated composite disease activity measure and tracking of the calculated patient data. No available apps meet the aforementioned criteria along with inclusion of 28 tender and swollen joint counts. Thus, current apps fall into two categories: simple calculators for rheumatologists and data tracking tools for people with RA. The latter do not uniformly collect data using validated composite disease activity measures. Apps that were rated highly according to the MARS tended to collect only patient-reported outcomes.

The rheumatology professional workforce is inadequate to meet current population rheumatology health care needs. Since demand for care is predicted to increase, adoption of different models of care provision will be necessary [18,35,36]. These are likely to include telehealth and an increased emphasis on participatory health care where people with RA are active agents in the management of RA. Developing apps that are attractive, engaging, simple to use, and having functionalities relevant to the clinical management of the health condition will require collaboration between rheumatologists, people with RA, app developers, and health systems, and due consideration of local regulatory environment, health service delivery, and user experience [22]. Once apps are developed, assessment of the validity and accuracy of self-performed joint counts will be required along with demonstration of equivalent health outcomes for people with RA whose care is provided with a mixed face-to-face and telehealth approach.

## Abbreviations

ACR: American College of Rheumatology
Apps: applications
CDA: composite disease activity
CDAI: clinical disease activity index
DAS28: disease activity score 28 joints
EULAR: European League Against Rheumatism
HAQ: health assessment questionnaire
ICC: intraclass correlation coefficient
Lab: laboratory data
MARS: mobile app rating scale
mHealth: mobile health
NZ: New Zealand
P: pain
PAS: patient activity scale
PAS-II: patient activity scale II
PhG: physician global assessment of disease activity
PRISMA: Preferred Reporting Items for Systematic Reviews and Meta-Analyses
PtG: patient global assessment of disease activity
RA: rheumatoid arthritis
RA info: rheumatoid arthritis information
RAPID-3: routine assessment of patient index data
SDAI: simple disease activity index
T: tender
28TJC: 28 tender joint count
28SJC: 28 swollen joint count
